# Pathomechanisms of Diabetic Kidney Disease

**DOI:** 10.3390/jcm12237349

**Published:** 2023-11-27

**Authors:** Satyesh K. Sinha, Susanne B. Nicholas

**Affiliations:** 1 Department of Medicine, David Geffen School of Medicine, University of California, Los Angeles, CA 90095, USA; sksinha@mednet.ucla.edu; 2 College of Medicine, Charles R Drew University of Medicine and Science, Los Angeles, CA 90059, USA

**Keywords:** metabolic, hemodynamic, renin angiotensin aldosterone system, inflammatory and fibrotic factors, mineralocorticoid receptor, osteopontin, targeted therapies

## Abstract

The worldwide occurrence of diabetic kidney disease (DKD) is swiftly rising, primarily attributed to the growing population of individuals affected by type 2 diabetes. This surge has been transformed into a substantial global concern, placing additional strain on healthcare systems already grappling with significant demands. The pathogenesis of DKD is intricate, originating with hyperglycemia, which triggers various mechanisms and pathways: metabolic, hemodynamic, inflammatory, and fibrotic which ultimately lead to renal damage. Within each pathway, several mediators contribute to the development of renal structural and functional changes. Some of these mediators, such as inflammatory cytokines, reactive oxygen species, and transforming growth factor β are shared among the different pathways, leading to significant overlap and interaction between them. While current treatment options for DKD have shown advancement over previous strategies, their effectiveness remains somewhat constrained as patients still experience residual risk of disease progression. Therefore, a comprehensive grasp of the molecular mechanisms underlying the onset and progression of DKD is imperative for the continued creation of novel and groundbreaking therapies for this condition. In this review, we discuss the current achievements in fundamental research, with a particular emphasis on individual factors and recent developments in DKD treatment.

## 1. Introduction

With the rising global prevalence of diabetes mellitus (DM) [1,2], diabetic kidney disease (DKD) remains the leading cause of chronic kidney disease (CKD), often progressing to kidney failure requiring replacement therapy in the form of dialysis or kidney transplantation [3,4,5]. Additionally, DKD is closely associated with heightened cardiovascular risks, including coronary artery disease, heart failure, sudden cardiac death, and increased morbidity and mortality [6,7]. The impact of DKD on both individuals with DM and healthcare systems is substantial. The development and progression of DKD are believed to stem from the complex interplay of metabolic, hemodynamic, inflammatory, and fibrotic factors, which are frequently disrupted in DM [8,9,10]. Dysfunction in these factors may be interlinked, influencing gene regulation, activating transcription factors, and affecting molecular pathways [8,10]. These interactions lead to functional and structural changes culminating in the clinical manifestations of DKD, characterized by escalating albuminuria and declining renal function [8]. While conventional approaches like optimal blood pressure control through renin–angiotensin–aldosterone system (RAAS) blockade and glycemic control have demonstrated efficacy in slowing down the progression of DKD, they do not halt or reverse the condition [11]. Therapies directed at pathways associated with metabolic changes, renal inflammation, fibrosis, and oxidative stress have displayed notable benefits in animal models. Consequently, several of these agents have undergone examination in clinical trials involving human subjects, yielding varied outcomes [11]. This review concentrates on several of the pathways and factors contributing to renal injury associated with DKD, drawing from a range of experimental nephropathy models, including published studies conducted by the author’s research group. For instance, our laboratory initially demonstrated for the first time that osteopontin (OPN) modulates angiotensin II (AngII)-induced inflammation, oxidative stress, and fibrosis in OPN-null mice in the heart [12] and then in the kidney [13]. We later showed that OPN plays a critical role in the development of DKD [14]. Our research was then used as the basis to reveal that the proteolytically cleaved fragment of OPN, known as N-terminal OPN (nt-OPN), exhibits a more substantial role in this pathology [15], and these findings are discussed. In addition to a description of established and novel pathways to DKD, we discuss existing therapies that target specific pathways involved in DKD.

### Factors That Promote DM and DKD

**Figure 1:**
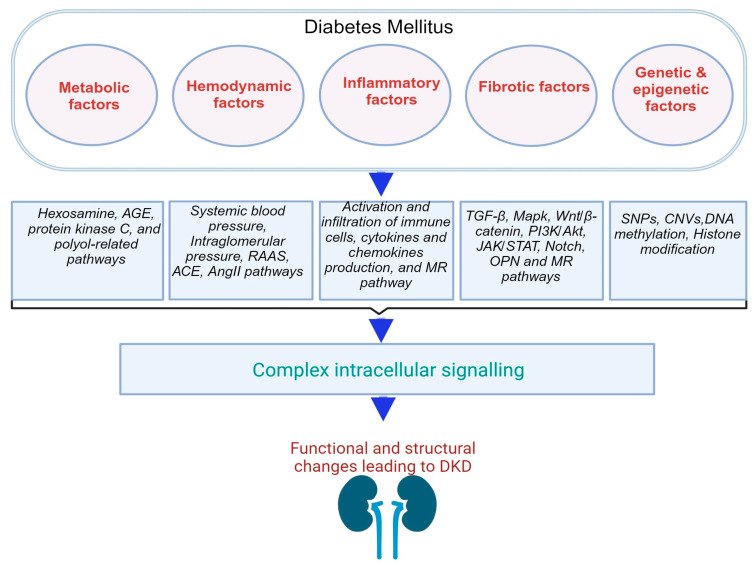
Factors that promote diabetes and DKD. The diagram illustrates that the progression of DKD encompasses a range of factors, including metabolic, hemodynamic, inflammatory, fibrotic processes, and genetic and epigenetic factors. These components impact various pathways that oversee intricate intracellular signaling networks, ultimately resulting in both functional and structural alterations within the kidney. Abbreviations: DKD: diabetic kidney disease, MR: mineralocorticoid receptor, AGE: advanced glycation end products, ACE: angiotensin-converting enzyme, RAAS: renin–angiotensin–aldosterone system, TGF-β: transforming growth factor-β, MAPKs: mitogen-activated protein kinases, PI3K/AKT: phosphatidylinositol-3-kinase/Ak strain transforming, JAK/STAT: Janus kinase/signal transducers and activators of transcription.

The development of DKD involves multiple factors, encompassing structural, metabolic, hemodynamic, inflammatory, and fibrotic processes in addition to genetic factors. These contribute collectively to the gradual deterioration of kidney function (Figure 1).

## 2. Metabolic Factors

**Figure 2:**
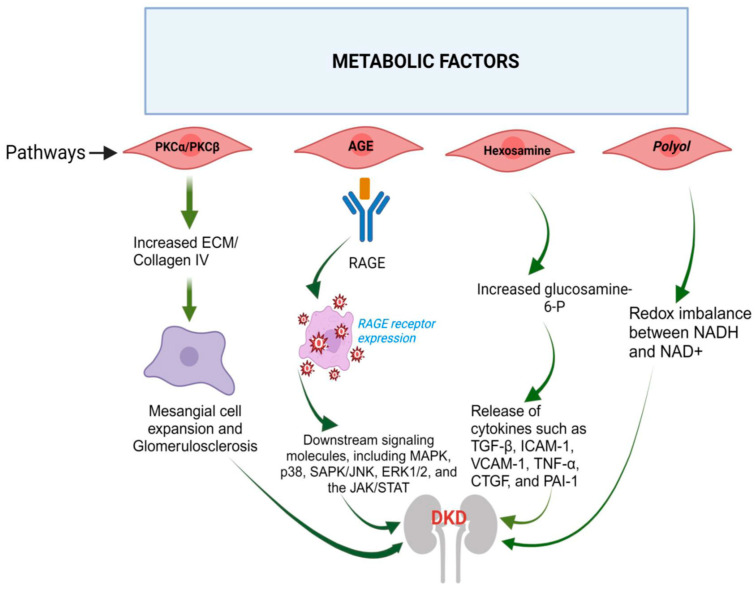
Role of metabolic factors in the development of DKD. The diagram depicts the impact of metabolic disruptions in hyperglycemia on various pathways, namely PKC, AGE, hexosamine, and the polyol pathway. The PKC pathway is linked to heightened production of ECM, especially collagen IV, leading to the expansion of mesangial cells and the development of glomerulosclerosis. The interaction between AGE and RAGE triggers downstream signaling molecules, including MAPK, p38, SAPK/JNK, ERK1/2, and JAK/STAT. The hexosamine pathway is responsible for generating glucosamine-6-p, which in turn promotes the release of cytokines such as TGF-β, ICAM-1, VCAM-1, TNF-α, CTGF, and PAI-1. The polyol pathway results in a redox imbalance between NADH and NAD^+^ in diabetes mellitus. Collectively, these processes contribute to the progression of DKD. Abbreviations: DKD: diabetic kidney disease, glucosamine-6-P: glucosamine-6-phosphate, PKC: protein kinase C, AGE: advanced glycation end products, ECM: extracellular matrix, RAGE: receptor for advanced glycation end products, SAPK/JNK: stress-activated protein kinases/Jun amino-terminal kinases, ERK1/2: extracellular signal-regulated kinase 1/2, JAK/STAT: Janus kinase/signal transducers and activators of transcription, TGF-β: transforming growth factor- β, ICAM-1: intercellular adhesion molecule 1, VCAM-1: vascular cell adhesion molecule 1, TNF-α: tumor necrosis factor alpha, CTGF: connective tissue growth factor, PAI-1: plasminogen activator inhibitor 1.

The early alterations in DKD are initiated by metabolic factors, particularly elevated blood glucose levels or hyperglycemia [16]. The harm caused by hyperglycemia can arise from direct tissue modifications or byproducts generated during glucose metabolism [16]. A visual representation of the disrupted metabolic pathways driving DKD development in individuals with DM is depicted in Figure 2 and discussed below.

Elevated blood glucose levels trigger the activation of pathways such as hexosamine, polyol, advanced glycation end products (AGE), and protein kinase C (PKC) [17]. This activation results in increased production of reactive oxygen species (ROS) and higher levels of mitogen-activated protein kinase (MAPK), JAK signal transducers and activators of transcription, and NF-κB [18]. These factors collectively contribute to the development of inflammation and fibrosis. MAPK is associated with the production of the extracellular matrix (ECM) and injury to podocytes [19]. NF-κB prompts the generation of adhesion molecules and cytokines, including monocyte chemoattractant protein (MCP-1), IL-6, and tissue necrosis factor (TNF)-α [20]. Additionally, ROS directly inflicts damage on cellular structures by oxidizing various lipids, nucleic acids, and proteins [21]. Here we discuss each of these pathways in detail.

### 2.1. Pathways Affected by Metabolic Disturbances in Hyperglycemia

#### 2.1.1. PKC Pathway

PKC is an intracellular signaling molecule that has the potential to slow down, or even halt the advancement of diabetic complications, including DKD [22,23]. The PKC family encompasses several isoforms including: α, β I, β II, γ, and δ [24], and the role of these isoforms in the development of DKD has been further clarified through the use of experimental rodent models [24]. Also, the elevated expression of mRNAs associated with the PKC-MAPK pathway is linked to glomerular lesions in patients with diabetic nephropathy (DN) [25]. Findings indicate that the PKC pathway is associated with the advancement of DKD by narrowing small blood vessels in the kidney and thus affecting the function of glomeruli [26]. In the mouse model of DM, phenylephrine-induced contraction of interlobar arteries is significantly augmented, inducing interlobar artery dysfunction which further diminishes blood flow within the glomerulus, facilitating the progression of DKD. Treatment with rottlerin, a calcium-independent PKC inhibitor, mitigates excessive basal contraction [27,28]. Similar findings were observed in diabetic Zucker rats, where the nonselective PKC agonist phorbol-12,13 dibutyrate reduced renal cortical blood flow and raised mean arterial pressure [29]. These outcomes highlight the association of PKC activation with decreased blood flow, heightened renal perfusion pressure, glomerulosclerosis, and reduced glomerular filtration.

The role of PKC in vascular permeability in DKD is particularly pronounced in the glomeruli, and PKC-α and PKC-β have emerged as significant players in maintaining glomerular filtration function [30,31]. Studies revealed that PKC-α and PKC-β were upregulated in diabetic mice, and the deletion of NOX4 reversed their overexpression and subsequently normalized nephrin expression [31], which is crucial for podocytes in maintaining slit diaphragm integrity for optimal filtration. Further, mediation of the ras homolog gene family, member A (RhoA) downstream of C3aR in endothelial cells by PKC resulted in kidney damage and increased blood vessel permeability [32]. 

In DKD, expansion of the glomerular basement membrane (GBM) and the accumulation of ECM are notable features [33]. In this pathology, the elevated activation of PKC was observed along with the increased production of ECM proteins such as fibronectin, laminin, and types I, III, and IV collagen in renal glomerular mesangial cells [34,35,36]. This effect is replicated by PKC activators like phorbol 12-myristate 13-acetate and oleoyl acetyl glycerol, a cell-permeable diacylglycerol analog [34]. The role of PKC activation in ECM protein synthesis and ECM degradation is further underscored in various studies. For instance, hyperglycemia promotes the secretion of hyaluronan (HA), a key ECM element, and PKC-β inhibition curbs HA secretion by reducing HAS2 mRNA expression [37]. A specific PKC inhibitor (GF 109203X) counteracts hyperglycemia-induced elevation in collagenous and total ECM protein synthesis and reduces human endothelial cell gelatinase activity [38]. PKC-α and PKC-β activation is also elevated in mesangial cells under high glucose concentrations, paralleling fibronectin, and IV collagen synthesis [39,40]. LY333531, a specific PKC-β inhibitor, halts ECM component expression in mesangial cells in hyperglycemia [40]. Global PKC-α/PKC-β double knockout mice exhibit diminished ECM component expression alongside reduced albuminuria development [24]. PKC-δ-/- and PKC-ε-/- streptozotocin (STZ)-induced diabetic mice, and mice with inhibited PKC-δ, reveal reduced ECM protein synthesis in mesangial cells [41,42,43]. Notably, PKC-ε-/- mice exhibit a profibrotic phenotype solely in the kidney [42]. The activation of PKC-δ, instead of PKC-α or PKC-β, is heightened in mesangial cells under high glucose conditions [43]. The membrane association of PKC-ζ, demonstrated to be hindered by rosiglitazone, is linked to reduced collagen IV expression in mesangial cells [44]. 

Transforming growth factor β1 (TGF-β1) plays a significant role in the accumulation of GBM and ECM in DKD [45,46] along with heightened activation of PKC [22,47,48]. Inhibition of PKC-α and PKC-β leads to a reduction in the expression of TGF-β1 and connective tissue growth factor (CTGF) in both glomerular endothelial cells and mesangial cells [22,39,40,47,49,50]. Mice with a knockout of Akr1b3 show inhibited PKC activation, resulting in decreased ECM accumulation and glomerular hypertrophy [51]. Homologous genes NOX2, NOX4, and NOX5 suppress the activation of PKC-α and PKC-β, leading to reduced levels of ROS, TGF-β1 expression, and MCP-1 in conditions of hyperglycemia. This, in turn, helps prevent ECM accumulation and thickening of the GBM in mesangial cells and podocytes [52,53,54]. 

Extracellular signal-regulated kinase (ERK) also plays a role in ECM accumulation. PKC-δ expression is increased under high glucose conditions, and its inhibition through rottlerin suppresses ERK expression and TGF-β1 responsiveness, blocking the fibrotic response in mesangial cells [55]. Conversely, PKC-β inhibition reduces ERK1 and ERK2 expression in mesangial cells [56]. Moreover, the involvement of phospholipase C-γ1 (PLC-γ1) in DKD development is highlighted; PLC-γ1 inhibition curbs PKC-β-induced protein kinase B (AKT) S473 phosphorylation and prevents collagen I upregulation [57]. In summary, PKC overexpression is implicated in ECM accumulation, primarily arising from mesangial cells; however, findings on PKC isoforms yield mixed results [24,43,58]. 

These findings indicate that the pursuit of a therapeutic approach involving isoform-specific inhibitors for targeting the PKC pathway holds promise. However, robust support for this potential treatment avenue necessitates well-designed, large-scale, and long-term clinical studies.

#### 2.1.2. AGEs/Receptor for Advanced Glycation End Products (RAGE) in DM

AGEs are enduring post-translational protein modifications that arise from the spontaneous interaction with glucose and associated metabolites. The AGEs cause harmful effects in two ways: firstly, by directly trapping and linking proteins together, and secondly, by attaching to a receptor on the cell surface [59,60]. Although AGEs can interact with various receptors, the precise interactions and their roles in cellular responses are not fully understood [61,62]. AGEs can influence cell functions by binding to toll-like receptors, scavenger receptors, G-protein-coupled receptors, and pattern recognition receptors [61,63]. Among these, the most crucial cell surface receptor for AGEs is RAGE, a member of the immunoglobulin superfamily. It was initially identified due to its ability to bind with AGEs [64,65]. Notably, RAGE is unique in its capacity to recognize three-dimensional structures rather than specific amino acid sequences. RAGE is considered a pattern-recognition receptor because of its ability to identify the structural characteristics of its ligands [64,65,66].

The full-length RAGE (fl-RAGE) protein, the most prevalent form, is composed of three domains: an extracellular domain with a V-type N-terminal domain and two C-type (C1 and C2) immunoglobulin domains, a transmembrane domain, and a cytosolic domain rich in charged amino acids [64,65]. The extracellular V-type domain primarily interacts with potential ligands outside the cell, while the cytoplasmic tail is crucial for intracellular signaling and serves as a scaffold for initiating signal transduction [67]. Also, the primary RAGE transcript undergoes alternative splicing and proteolytic cleavage of fl-RAGE to produce truncated RAGE isoforms, a process governed by unknown pathways [68,69,70]. The activation of RAGE by AGEs leads to an increase in RAGE receptor expression, creating a positive feedback loop where ligand-stimulated RAGE amplifies and sustains its own activity [71,72]. 

In DM, elevated circulating AGEs interact with its receptor, RAGE, and induce downstream signaling molecules, including MAPK, p38, stress-activated PKC-Jun N-terminal kinase (SAPK/JNK), Ras-mediated ERK1/2, and the JAK/STAT pathway. These pathways subsequently lead to the prolonged activation of transcription factors such as NF-κB, STAT3, HIF-1α, and AP-1 [62,73,74]. Hence, intervening in DKD by targeting RAGE along with its ligands to mitigate oxidative stress and chronic inflammation is viewed as an additional strategy. Nevertheless, a clinical trial involving an advanced AGE inhibitor, while demonstrating protective effects against kidney injury [75], was halted due to severe side effects [76].

#### 2.1.3. Hexosamine Pathway

The hexosamine biosynthetic pathway is thought to be involved in the progression of insulin resistance and the emergence of vascular complications in DM. It holds significance in the synthesis of proteoglycans, glycolipids, and glycoproteins [77]. This pathway involves converting fructose-6-phosphate (fruc-6-P) into glucosamine-6-phosphate (glucN-6-P) using glutamine as the amino donor, mediated by the rate-limiting enzyme, glutamine: fructose-6-phosphate-amidotransferase (GFAT). Subsequently, glucN-6-P is rapidly utilized in generating uridine-5-diphosphate-N-acetylglucosamine (UDP-N-acetylglucosamine), a precursor required for synthesizing amino sugars needed for glycoproteins, proteoglycans, glycosaminoglycans, and glycolipids [77,78,79]. Elevated blood sugar levels foster DM complications by increasing fruc-6-P concentration, driving it into the hexosamine biosynthetic pathway [80,81]. Concomitantly, heightened glucose concentrations induce metabolic pathways that culminate in the release of cytokines such as TGF-β, ICAM-1, VCAM-1, TNF-α, CTGF, and plasminogen activator inhibitor (PAI)-1, which play roles in diverse diabetic complications [82,83]. For instance, TGF-β1 significantly contributes to DKD [77]. The critical role of PAI-1 in DKD was demonstrated in our own work in which PAI-1 was shown to regulate TGF-β1 expression, and deletion of PAI-1 reduced TGF-β1 and retarded the development of DN [84]. Cellular glucose uptake also channels a relatively larger portion of glucose to glycogenesis, glycolysis, and pentose phosphate metabolism. Additionally, about 2–3% of glucose molecules are directed into the hexosamine biosynthetic pathway [83,85]. Blocking the hyperglycemia-induced transcription of cytokines is achieved by inhibiting the rate-limiting enzyme, GFAT, which effectively prevents potential diabetic complications arising from this pathway [77,80,86]. It was demonstrated that the heightened production of mitochondrial superoxide due to hyperglycemia leads to an elevation in hexosamine synthesis and the O-glycosylation of specificity protein 1 (Sp1). This, in turn, triggers the activation of genes that play a role in the development of diabetic complications [87].

#### 2.1.4. Polyol Pathway

The polyol pathway involves two enzymatic reactions [88,89]. The initial reaction is the reduction of glucose to sorbitol, facilitated by the enzyme aldose reductase (AR). This step is considered the rate-limiting process in the pathway and results in the conversion of NADPH to NADP+ [89,90]. The subsequent reaction transforms sorbitol into fructose, catalyzed by sorbitol dehydrogenase, generating NADH from NAD+. Thus, the end products of the polyol pathway include sorbitol, fructose, and NADH [89]. Therefore, in DM, this pathway is believed to be a primary contributor to the redox imbalance between NADH and NAD+ [89,91,92]. 

In a murine model of STZ-induced DM, the absence of the *AR* gene significantly improved the progression of early DKD indicators [51]. In this study with diabetic AR-null mice, there was complete inhibition of DM-induced ECM build-up and excessive collagen IV production. Additionally, deficiency of AR led to a complete or partial blockade of the DM-induced activation of renal cortical PKC, TGF-β1, and glomerular hypertrophy. In diabetic *AR*-null mice, there was a significant decrease in urine albumin excretion [51]. Studies have revealed the potential of AR inhibitors (like pyrogallol) in alleviating DM and a variety of AR inhibitors have undergone testing and assessment [93]. In contrast to the experimental evidence, AR inhibitors only have a partial effect in preventing DKD in patients [92]. 

In hyperglycemic conditions, the activation of the polyol pathway results in an increase in fructose levels, potentially worsening DM, and its related complications [89,94]. Fructose has dual effects: it can chemically glycate proteins, leading to their dysfunction [95]; additionally, its metabolism by fructokinase, which consumes ATP, bypasses the regulatory mechanisms of the glycolytic pathway [96,97]. This can lead to an overproduction of acetyl-CoA and depletion of ATP, further contributing to protein functional impairment [98,99].

Moreover, the kidney, being abundant in mitochondria [100], implicates the redox reactions and energy metabolism within these organelles in the onset of metabolic disorders in the kidney, including DKD [101]. On a molecular level, altered glucose metabolism leads to a notable redox imbalance in the NADH/NAD+ ratio [102,103], which may emerge as a distinctive mechanism contributing to diabetic kidney injury [104,105]. This is due to the fact that electrons generated from the breakdown of glucose and other nutrients like fatty acids and amino acids are retained in NADH, utilizing NAD+ as the electron acceptor [106,107]. Consequently, a prominent characteristic of DM is an excess of NADH and a deficiency of NAD+ [89,103]. Several pathways have been investigated for their involvement in NAD consumption. These include the Poly ADP Ribosylation Pathway [108], Sirtuins Pathway [109], CD38 Pathway, and NAD Kinase Pathway [110]. The significance of the Poly ADP Ribosylation Pathway in the development of DM has been established through studies in murine models, where the absence of this enzyme prevents the onset of DM [111]. This highlights the harmful consequences of NAD+ depletion in diabetes. Additionally, research has demonstrated that CD38-driven NAD+ deficiency is responsible for organ fibrosis and dysfunction in the kidneys of diabetic individuals [112]. 

## 3. Hemodynamic Factors

DM may also lead to hemodynamic effects, which include increased systemic blood pressure [113] and increased intraglomerular pressure [114]. The elevation in glomerular capillary pressure gives rise to an increase in the glomerular filtration rate (GFR) of a single nephron, a phenomenon referred to as hyperfiltration [115,116,117]. In DKD, glomerular hyperfiltration is the first stage in the pathogenesis, leading to progressive albuminuria, declining GFR, and finally kidney failure or end-stage renal disease (ESRD), although the mechanism is not fully understood [115]. The escalation in intraglomerular pressure arises from heightened tone in the efferent arteriole coupled with a reduction in tone in the afferent arteriole [117]. The exact mechanism behind this process is not definitively established, yet two main theories have emerged.

One perspective posits that hyperfiltration is driven by molecules present in the circulation that exert their effects predominantly within the glomerulus [118]. Multiple mediators have been suggested as agents that could amplify intraglomerular pressure by increasing tone in the efferent arteriole and diminishing tone in the afferent arteriole. Elevated resistance in the efferent arteriole can arise due to heightened levels of AngII, thromboxane A2, endothelin (ET) 1, and ROS [117]. Conversely, diminished resistance in the afferent arteriole can be initiated by a decrease in the availability of nitric oxide, an increase in cyclooxygenase-2 prostanoids, activation of the kallikrein-kinin system, atrial natriuretic peptide, angiotensin 1–7, and an increase in insulin [117]. 

**Figure 3:**
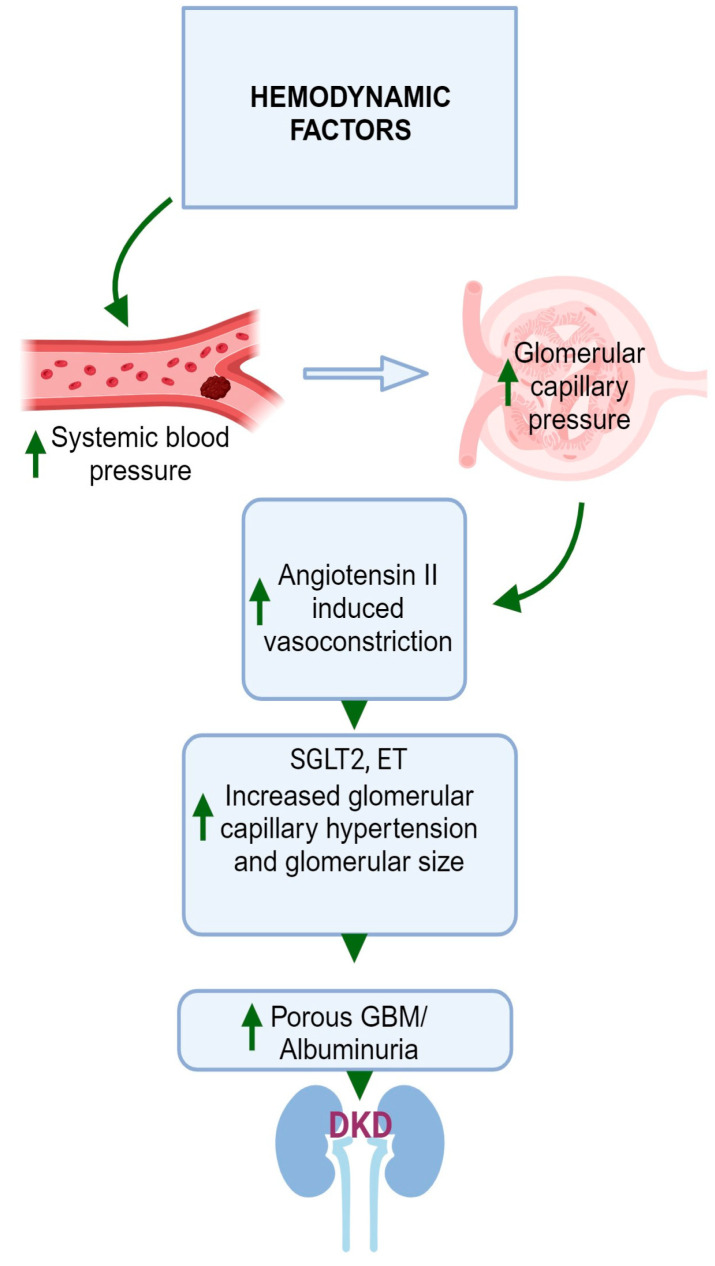
Role of hemodynamic factors in the development of DKD. The figure demonstrates the process by which diabetes gives rise to hemodynamic changes, characterized by elevated systemic blood pressure and heightened intraglomerular pressure. These factors collectively encourage angiotensin II-induced vasoconstriction. The amplified glomerular capillary pressure and increased glomerular size led to a more permeable GBM, consequently resulting in albuminuria. These combined effects contribute to the development of DKD. Abbreviations: SGLT2: sodium–glucose cotransporter 2, ET: endothelin, DKD: diabetic kidney disease, GBM: glomerular basement membrane.

However, another perspective suggests that tubular mechanisms play a more pivotal role in driving intraglomerular hypertension [119]. The activation of glucose transport pathways in the proximal tubule at the early stages of DM triggers enhanced reabsorption of both glucose and sodium in the proximal nephron [119]. Consequently, the delivery of sodium to the distal nephron decreases. This prompts a response known as tubuloglomerular feedback, leading to dilation of the afferent arteriole and constriction of the efferent arteriole [119]. The sodium–glucose cotransporter 2 (SGLT2) is a postulated mechanism of glomerular hyperfiltration. SGLT2 increases the reabsorption of glucose in the proximal tubules, thereby reducing the delivery of sodium chloride to the macula densa [120]. As a result, tubuloglomerular feedback is reduced, afferent arterioles are dilated, and AngII is increased in efferent arterioles, resulting in vasoconstriction [121,122]. These effects increase glomerular perfusion and intraglomerular pressure, leading to glomerular hyperfiltration. Moreover, an increase in insulin on its own can enhance sodium and glucose transport in the proximal tubule, thereby inciting tubuloglomerular feedback. As highlighted earlier, insulin can also directly reduce tone in the afferent arteriole. As a result, insulin can exert both direct and indirect influences that contribute to the occurrence of hyperfiltration. Figure 3 illustrates a graphical depiction of the altered hemodynamic factors contributing to the development of DKD in individuals with DM. 

### 3.1. RAAS Pathway

The intrarenal hemodynamic function is significantly impacted by the RAAS [123]. This system heightens oxidative stress and triggers pro-inflammatory pathways. These processes lead to glomerular hypertrophy, a feature that manifests in the early stages of DKD and marks the initiation of a profibrotic cascade [124]. The favorable effects of RAAS blockers on retarding the progression of DKD have been extensively documented, as evidenced by numerous studies [125]. This effect holds true despite the presence of a relatively low systemic renin state in individuals with DKD. This phenomenon is believed to be a manifestation of the activated local renin system within the kidneys or an increased sensitivity to AngII at the intrarenal level [126]. The varying stages of kidney disease and diverse assessment methods used (including measuring renin activity, protein levels, RNA expression, serum potassium, and bicarbonate concentrations, and employing techniques like immunohistochemistry and fluorescence) have resulted in conflicting clinical data regarding the measurement of RAAS activation in DKD [127,128]. Conversely, findings from experimental models have consistently demonstrated elevated RAAS activation in DKD [129,130,131,132]. This activation of the RAAS encompasses all elements and stages of the cascade, and it occurs in a localized manner, operating in a paracrine fashion. The RAAS cascade commences with the synthesis of prorenin within the juxtaglomerular cells, which is subsequently cleaved into renin [133]. Renin then catalyzes the cleavage of angiotensinogen (AGT) to generate angiotensin I (AngI). This AngI is further transformed by the angiotensin-converting enzyme (ACE) into the octapeptide known as AngII. 

RAAS blockade has been observed to have favorable effects on renal outcomes predominantly in placebo-controlled clinical trials [125,134]; however, the favorable effect may potentially be explained by the blood pressure-lowering effect of RAAS blockade [135]. Significant trials have demonstrated distinct advantages of AngII receptor blockers (ARB) for individuals with DKD [136,137]. It is theoretically considered that dual blockade is more effective than single blockade because ACEIs and ARBs act on different sites within the RAAS [138]. Early studies suggested a combination of ACE inhibitors and ARB may provide additional benefits in diabetic nephropathy [139,140], however, this combination is not clinically recommended due to complications of hyperkalemia and acute kidney injury. 

### 3.2. Prorenin and Renin

In individuals with DM, approximately 95% of circulating renin is in the form of prorenin. The precise mechanism through which prorenin contributes to the development of DKD is not well known [141]. Prorenin binds to the prorenin receptor (PRR) and, independently of AngII, initiates intracellular signaling. This signaling leads to the activation of mitogen-activated protein (MAP) kinases ERK1/2, resulting in the upregulation of proteins such as TGF-β1, PAI-1, collagens, fibronectin, and cyclooxygenase-2 [142,143,144,145,146]. This suggests that elevated prorenin levels might contribute to the progression of DKD by stimulating PRR and prompting the synthesis of pro-fibrotic proteins [133]. Notably, in experiments with transgenic rats that overexpress human PRR, these rats develop proteinuria and exhibit a gradual onset of glomerulosclerosis and kidney damage independent of AngII [129]. Intriguingly, inducing overexpression of prorenin alone does not lead to glomerulosclerosis, hinting that prorenin may enhance fibrosis rather than directly causing it [130,147]. Additionally, prorenin plays a role in generating AngI. When prorenin binds to PRR, it induces a conformational change that involves the unfolding of the peptide from the enzymatic cleft, making the cleft accessible to AGT and allowing the generation of AngI [148,149]. In patients with DM, elevated plasma prorenin levels were observed [150], alongside reduced plasma renin levels compared to normal healthy subjects [126]. The coexistence of elevated prorenin levels and heightened renal PRR expression in DM [151] implied a potential involvement of this receptor in the development of DKD.

In the early stages of DM, there is a notable increase in the expression of renin mRNA within the proximal tubule [131]. Beyond its traditional role in enhancing the synthesis of AngII, renin has a direct effect on stimulating the production of TGFβ, a cytokine associated with fibrosis [143]. Renin binds to its specific receptor on the cell surface of mesangial cells, resulting in cell hypertrophy and an increased efficiency of AGT cleavage by renin. This process unmasks the catalytic activity of prorenin [152]. Interestingly, the renin receptor has also been found in the sub-endothelium of renal arteries, suggesting that renin has a novel receptor-mediated action that could potentially contribute to renal fibrosis [153]. Moreover, in podocytes, elevated glucose levels have been demonstrated to lead to increased AngII generation. This occurs by boosting renin mRNA expression, accompanied by a simultaneous increase in the PRR levels, ultimately enhancing the conversion of AGT to Ang I [154].

### 3.3. ACE

The most compelling evidence supporting the existence of intrarenal renin production and activity in DM comes from animal models of spontaneous DM or those induced by STZ [131]. Interestingly, despite having either suppressed or normal levels of plasma renin activity, DM leads to increased renin mRNA and protein levels within the kidneys, particularly in the proximal tubules [131]. In rats with DM, there is a notable reduction in total renal ACE activity, accompanied by a specific redistribution of ACE within the diabetic kidneys [155]. While ACE activity in the proximal tubules is decreased, staining intensity for ACE is intensified in diabetic glomeruli and the renal vasculature. This observation suggests a potential role for glomerular ACE in mediating nephron injury, potentially by enhancing the local formation of AngII within the glomerulus [155].

### 3.4. AngII

As mentioned earlier, DKD is associated with an upsurge in intrarenal AngII generation, despite the systemic suppression of the RAAS. The adverse effects of this elevation in AngII extend beyond hemodynamic alterations and encompass insulin resistance, cell growth stimulation, and damage to the tubules. One of the pivotal roles of AngII in DKD is its association with volume expansion achieved through water and sodium reabsorption.

AngII activates the luminal membrane Na^+^–H^+^ antiporter by stimulating an inhibitory G protein, which in turn reduces cyclic AMP generation. This reduction minimizes the typical suppressive influence of cyclic AMP on Na^+^–H^+^ exchange [68,156]. AngII also stimulates phosphatidylinositol turnover, leading to the production of PKC [156,157]. Moreover, it enhances the secretion of aldosterone from the adrenal cortex, further promoting sodium transport in the cortical collecting tubule [158]. In the proximal tubule, AngII inhibits proteinase activity and induces mesangial cell expansion by decreasing the activity of the plasminogen activator. Additionally, AngII leads to the upregulation of TGF-β1 and the release of vascular endothelial growth factor from glomerular epithelial and mesangial cells, both of which contribute to mesangial matrix expansion [159]. Renal fibroblasts express AngII type 1 (ATI) receptors and respond to AngII stimulation by proliferating, expanding the matrix, and synthesizing fibronectin, primarily through a TGF-β-dependent mechanism [160].

Microinflammation within the glomeruli and tubulointerstitial regions, followed by the expansion of the ECM, are shared pathways in the progression of DKD. AngII activates inflammatory cells directly, causing chemotaxis, including the release of OPN, RANTES, and other proinflammatory mediators like MCP-1 and TGF-β. It also triggers the activation of various intracellular signaling pathways, including PKC, protein tyrosine kinases, MAPK, ERK, c-Jun amino-terminal kinase (JNK), p38 MAP kinase (p38 MAPK), and the activator protein-1 (AP-1). These factors are implicated in processes such as proliferation, differentiation, fibrosis, and inflammation [160]. 

Furthermore, AngII has been linked to insulin resistance [161]. It hinders insulin-mediated GLUT4 translocation in skeletal muscle by transiently activating ERK1/2, which inhibits insulin receptor substrate ½ (IRS-1/2). Additionally, AngII directly inhibits insulin sensitivity through nitration of AKT and induces tyrosine phosphorylation of IRS-1 via Janus kinase 2, associated with AT1 receptor stimulation. This phosphorylation attenuates insulin-induced activation of phosphatidylinositol-3-kinase, ultimately leading to reduced insulin sensitivity [162]. Interestingly, treatment with the AngII receptor blocker candesartan increases renal insulin receptor expression in STZ-induced diabetic rat models and insulin-resistant Zucker rats, regardless of insulin levels [163].

In human DN, components of the RAS undergo alterations in the kidney, revealing increased local AngII production, activation of tubular cells, and the induction of proinflammatory markers. This indicates that AngII plays a role in the renal inflammatory process, potentially providing insight into the molecular mechanisms responsible for the beneficial effects of RAS blockade [164]. 

## 4. Inflammatory Factors

In the progression of DKD, several interconnected pathways contribute to disease development, and inflammation serves as a crossroad in its pathogenesis. DM initiates inflammatory processes mediated by several factors like oxidative stress, AGEs, obesity, ischemia, and cellular damage [165]. These processes collectively generate inflammatory molecules, including NF-κB, caspases associated with the NLR family pyrin domain containing 3 (NLRP3) inflammasome, TNF-α, IL-1β, IL-6, and IL-18.

Araújo et al. examined the expression levels of various cytokines and chemokines, including CCL11, macrophage inflammatory protein-1α, IL-8, IL-4, IL-10, TNF-α, TNFR1, IL-1β, and IL-6, in renal biopsies taken from patients diagnosed with DN. Their results demonstrated an increased expression of cytokines and chemokines in DN, specifically highlighting elevated levels of IL-6, IL-1β, IL-4, and CCL11. The study concluded that CCL11 might exert a significant influence on the progression of interstitial inflammation in DN and contribute to the decline in estimated GFR in these patients [166]. 

According to a separate study utilizing renal biopsies, it has been suggested that in DN, the primary inflammatory agent driving renal inflammation is IL-1α, rather than IL-1β, which is released by renal tubular cells [167]. Additionally, levels of IL-1α in both the urine and plasma of DM patients have been linked to markers indicating injury in podocytes and proximal tubular epithelial cells [168]. 

Similarly, urinary IL-6 could be a valuable indicator for identifying DKD in DM patients, even in cases where there is no detectable urinary albumin excretion [169]. Elevated levels of IL-6 in the serum have been observed in individuals with DKD [170] and were linked with GBM thickness [171]. Also, the expression of IL-6 mRNA in glomerular and interstitial cells in individuals with DKD was associated with mesangial proliferation causing renal injury [172]. In patients with DM, urinary IL-6 was also connected to the progression of DKD [173].

Furthermore, TNF-α serves as a crucial transcriptional regulator for components of the NLRP3 inflammasome [174,175]. In patients with DKD, elevated levels of serum TNF-α and its receptor have been observed, and they are indicative of both renal decline and the likelihood of ESRD occurrence [176,177]. It has been demonstrated that inhibiting TNF-α can notably alleviate glomerular lesions in murine models of DM [178]. The TNF-α inhibition provides protective effects against tubulointerstitial nephritis by suppressing the NLRP3 inflammasome in DN rats [179].

The increase in AGEs has been directly associated with an elevated expression of NLRP3-related proteins, which have been proposed as mediators of CKD, potentially activating mesangial cells [180]. NLRP3-related proteins are present in macrophages and inflammasomes and have been associated with various inflammatory disorders. In several mouse models, reducing NLRP3 activity has been shown to slow down the progression of CKD in a dose-dependent manner. Additionally, inflammation can lead to the infiltration of neutrophils and macrophages, oxidation of lipoproteins, and deposition of immune complexes [181]. This persistent inflammation also results in increased production and deposition of amyloid A protein, which can serve as a marker of disease progression [182].

Moreover, the activation of the complement system significantly influences the progression of DKD. The activation of the complement system in DKD has been linked to mannose-binding lectins and the ficolin-associated activation of the lectin pathway within the complement cascade. Hyperglycemia results in higher levels of glycan and galactosamine-bound substances that are recognized by these receptors, leading to complement activation [183]. Ongoing research may yield new complement inhibitors that offer benefits to DKD patients; however, careful monitoring is crucial to evaluate any impact on susceptibility to infections or immune complex diseases.

**Figure 4:**
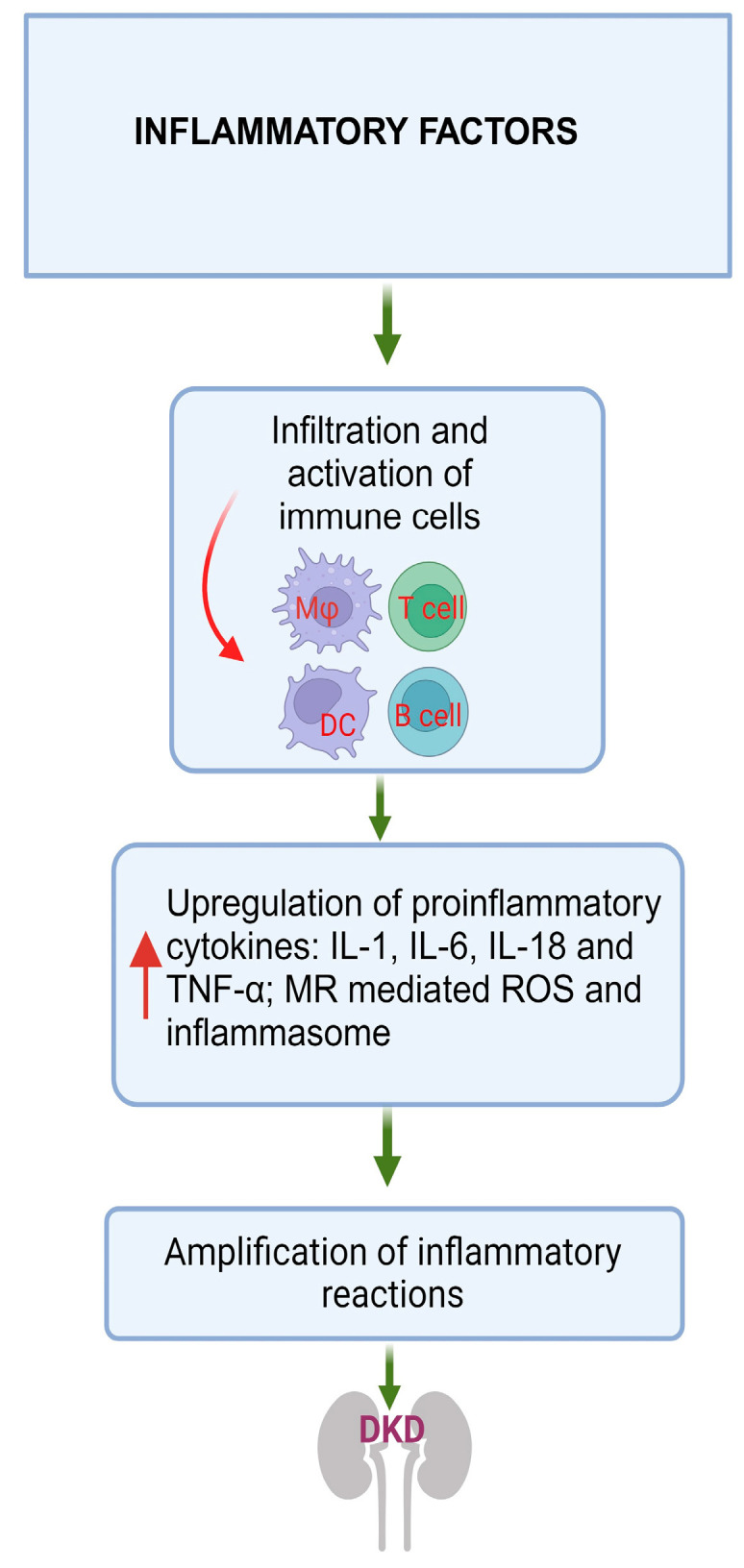
Role of inflammatory factors in the development of DKD. The diagram illustrates how inflammation plays a role in the development of DKD. In diabetes, there is an infiltration and activation of immune cells, including macrophages, T-cells, and B-cells, within renal tissue. This leads to an increased expression of proinflammatory cytokines like IL-1, IL-6, IL-18, and TNF-α. Consequently, this intensifies the inflammatory responses within the renal tissue, contributing to its damage. Abbreviations: DKD: diabetic kidney disease, IL: interleukin, TNF-α: tumor necrosis factor alpha, MR: mineralocorticoid receptor, ROS: reactive oxygen species, Mφ: macrophage, DC: dendritic cell.

In summary, DKD involves multiple intertwined pathways, with inflammation also playing a pivotal role in its development (Figure 4). This inflammation is initiated by various factors associated with DM and results in the production of inflammatory molecules and activation of the complement system, both of which contribute to the progression of the disease.

## 5. Fibrotic Factors

**Figure 5:**
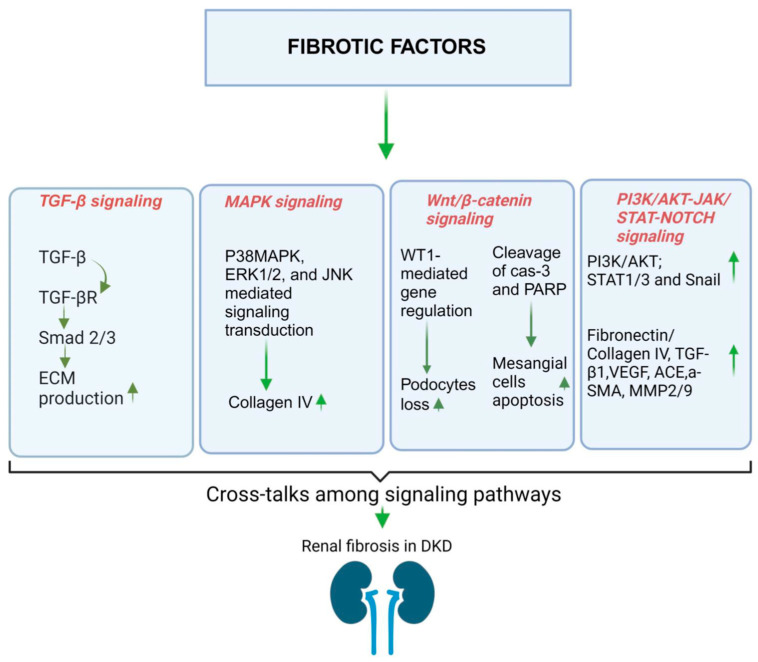
Role of fibrotic factors in the development of DKD. The diagram depicts the involvement of renal fibrosis in the development of DKD. This fibrotic process encompasses various pathways, including TGF-β, MAPK, Wnt/β-catenin, PI3K/AKT, JAK/STAT, and NOTCH signaling. Within the TGF-β signaling pathway, Smad 2/3 mediates the production of ECM. The MAPK pathway leads to an increase in signaling transduction mediated by P38MAPK, ERK1/2, and JNK, resulting in elevated collagen IV levels. The Wnt/β-catenin pathway contributes to podocyte loss and mesangial cell apoptosis through the regulation of WT-1-associated genes and the cleavage of cas-3 and PARP, respectively. The collective action of the PI3K/AKT-JAK/STAT-NOTCH signaling pathways promotes PI3K/AKT, STAT1/3, and Snail signaling, leading to the induced expression of fibronectin/collagen IV, TGF-β1, VEGF, ACE, α-SMA, MMP2/9. Ultimately, these molecules drive the process of renal fibrosis in DKD. Abbreviations: DKD: diabetic kidney disease, WT-1: Wilms tumor-1, a master regulator of gene expression in podocytes, TGF-β: transforming growth factor-beta, MAPK: mitogen-activated protein kinases, ERK1/2: extracellular signal-regulated kinase 1/2, JNK: c-Jun amino-terminal kinase, Cas-3: caspase-3, PARP: polyADP-ribose polymerase, PI3K/AKT: phosphatidylinositol-3-kinase/Ak strain transforming, STAT1/3: signal transducer and activator of transcription 1/3, JAK/STAT: Janus kinase/signal transducers and activators of transcription, Smad 2/3: suppressor of mothers against decapentaplegic 2/3, ECM: extracellular matrix, VEGF: vascular endothelial growth factor, ACE: angiotensin-converting enzyme, a-SMA: alpha smooth muscle actin, MMP2/9: matrix metalloproteinase2/9.

Renal fibrosis refers to an excessive buildup of ECM in the kidney, which can manifest as either glomerulosclerosis or tubulointerstitial fibrosis [184,185]. The main components of this fibrotic ECM consist of collagens I and III, known as fibrillar collagens, along with glycoproteins and proteoglycans [186]. The extent of fibrosis is closely linked to the decline in renal function and the progression towards ESRD [187]. Myofibroblasts play a pivotal role in facilitating ECM deposition due to their robust capacity to produce ECMs like collagens, thus contributing to the advancement of renal fibrosis [188]. In individuals with DM, there is a notable infiltration of activated myofibroblasts, a phenomenon generally not observed in a healthy physiological state [189]. However, there is ongoing debate regarding the role of myofibroblasts, particularly the process of epithelial to mesenchymal transition (EMT) where tubular epithelial cells transform into myofibroblasts in DKD [190]. Renal fibrosis constitutes a crucial pathological alteration in DKD and significantly elevates the mortality rate among late-stage DKD patients. The major signaling pathways implicated in renal fibrosis encompass the TGF-β, MAPK, Wnt/β-catenin, PI3K/AKT, JAK/STAT, and Notch pathways (Figure 5). Each of these pathways exerts a substantial influence on the buildup of ECM, the expression of collagen and fibronectin, as well as the secretion of other pertinent proteins [191].

### 5.1. TGF-β Signaling Pathway

It is widely recognized that TGF-β stands as the most significant profibrotic cytokine, serving as a primary driver of renal fibrosis in DKD [192,193]. DKD is linked to various detrimental factors, including elevated glucose levels, AGEs, hypertension, and dyslipidemia. These factors can trigger TGF-β signaling through both TGF-β-dependent and independent pathways [193]. High glucose levels amplify TGF-β signaling and boost the activity of specific elements involved in fibronectin transcription in various types of kidney cells, including mesangial, fibroblast, and proximal tubular cells [193,194]. This influence on TGF-β expression might be linked to a glucose-responsive element in the *Tgfb1* gene’s promoter [195]. Additionally, high glucose levels enhance the activity of TGF-β1 by increasing thrombospondin 1 (TSP1), which can activate latent TGF-βs [196,197]. Moreover, high glucose independently elevates the transcription of TGF-β receptor II in murine mesangial cells, even without the induction of TGF-β [198]. Thus, high glucose can activate TGF-β signaling during the development of DKD. 

In both patients and animal models with DKD, there is a substantial increase in the expression or activation of TGF-β ligands, TGF-β receptors, and the subsequent signaling mediators like Smad2 and Smad3 in glomeruli, tubules, and the tubulointerstitium [46,198,199,200,201]. This garnered substantial attention in the field, as evidenced by seminal studies demonstrating the protective effects of TGF-β antibodies [202]. TGF-β, operating through both Smad-dependent and -independent signaling pathways, assumes a pivotal role in myofibroblast activation. This leads to heightened production of ECMs and suppression of ECM turnover or degradation [203]. Ultimately, this results in an excessive buildup of ECM, a prominent hallmark of renal fibrosis. Moreover, it is evident that TGF-β also plays a role in inducing EMT, as described earlier, as well as endothelial to mesenchymal transition. These processes involve the conversion of local resident cells into those with more pro-sclerotic traits, thereby contributing to kidney injury and fibrosis [204].

The success of inhibiting TGF-β1 signaling in animal studies has prompted clinical investigations in DKD [205,206]. While pirfenidone, an antifibrotic agent, increased eGFR in a diabetic patient cohort [205], a placebo-controlled phase II study utilizing a humanized TGF-β1-specific neutralizing monoclonal antibody alongside renin–angiotensin system blockades did not succeed in slowing the progression of DKD in patients [206]. 

### 5.2. MAPK Signaling Pathway

MAPKs, a set of serine/threonine protein kinases, oversee a range of cellular functions and can become active under high-glucose conditions [191,207]. The primary subgroups within the MAPK family are P38/MAPK, ERK, and JNK, collectively playing a role in signal transduction during fibrosis [191]. Various studies have shown that modulating specific components of the MAPK signaling pathways can have significant effects on renal fibrosis in DKD. Increasing EphA1 expression was found to reduce phosphorylation of ERK1/2 and JNK, leading to the alleviation of renal fibrosis in mice models of DKD [208]. Additionally, the ERK1/2 MAPK pathway was identified as a crucial player in DKD, influencing fibrogenesis by regulating mesangial cell activities and ECM accumulation [209]. Inhibition of p38 MAPK showed promise in reducing the presence of phosphorylated p38 MAPK-positive cells, particularly in the early stages of fibrosis [210]. Targeting p38 MAPK exclusively was effective in alleviating renal fibrosis in an established fibrotic model [211]. Moreover, inhibiting JNK isoforms significantly delayed fibrosis progression by impeding the accumulation of collagen IV and α-SMA^+^ myofibroblasts in an animal model [212]. These findings collectively highlight the potential therapeutic targets within the MAPK pathways for managing renal fibrosis in DKD [191]. In an in vitro study, it was demonstrated that the activation of p38 MAPK and ERK in human podocyte cells through IL-17RA [134] while another study showed that pentosan polysulfate mitigated apoptosis and inflammation by suppressing the activation of the p38 MAPK pathway in human renal proximal tubular epithelial cells (HK 2) treated with high glucose [213]

### 5.3. Wnt/β-Catenin Pathway

The Wnt signaling pathway can be categorized into two primary pathways: the canonical β-catenin-dependent pathway and the non-canonical β-catenin-independent pathway [214]. The most extensively studied Wnt signaling pathway is the canonical Wnt/β-catenin pathway Recent studies proved that dysregulation of the Wnt signaling pathways participates in the occurrence and progression of type 2 DM by directly influencing the differentiation and proliferation of pancreatic β-cells and the secretion and action of insulin [215]. During CKD, the Wnt canonical signaling pathway becomes active, whereas it remains comparatively inactive in the normal adult kidney [216,217]. The Wnt canonical and non-canonical pathways have been identified as another key player in the progression of DKD [218,219]. Dysregulated Wnt signaling, which causes harm to podocytes [220,221,222] and mesangial cells [223,224,225], ultimately culminates in renal fibrosis [214]. An earlier study established that aberrant upregulation of the Wnt/β-catenin signaling pathway leads to podocyte damage and dysfunction [220]. The study reported that abnormal activation of the Wnt/β-catenin pathway hinders WT1 (a podocyte marker)-mediated gene expression, resulting in podocyte de-differentiation and mesenchymal transformation [221]. Conversely, blocking the Wnt/β-catenin pathway reinstates WT1-mediated gene regulation, thus preserving podocyte integrity [221]. Likewise, DM induces the production of Ras/Rac1-dependent superoxide, subsequently impeding Wnt proteins in mesangial cells [226]. In a murine model of DM, treatment with superoxide dismutase-conjugated propylene ethyl glycol demonstrated an improvement in the detrimental effects of free radicals on mesangial cells. High glucose initiates the activation of GSK-3β, resulting in the instability and degradation of β-catenin. This, in turn, induces mesangial cell apoptosis by promoting the cleavage of caspase-3 and polyADP-ribose polymerase (PARP) [224]. A previous study showed that transfecting mesangial cells with WNT4, WNT5a, or stable β-catenin (S33Y) can hinder the activation of GSK-3β and enhance the stability of nuclear β-catenin, thereby reducing the level of apoptosis in mesangial cells [225]. The use of medications such as simvastatin [227] and spironolactone [228] leads to increased secretion of Wnt5a protein in mesangial cells in patients with DKD. This heightened secretion of Wnt5a facilitates the translocation of β-catenin to the nucleus, thereby enhancing the protective effect against high glucose-induced inhibition of Wnt/β-catenin. This process ultimately safeguards glomerular mesangial cells and contributes to a beneficial outcome for individuals with DKD. Hence, examining the expression of Wnt proteins holds significant relevance for DKD. 

### 5.4. PI3K/AKT, JAK/STAT, and Notch Pathways

The PI3K/AKT signaling pathway assumes a central regulatory role in the progression of DKD. In DM, this pathway becomes activated in renal tubular cells, overseeing processes such as cell growth, EMT, and lipid metabolism [229,230]. Using a murine model of DKD, a recent study indicates that METTL14 (a core component of methyltransferase complex)-regulated PI3K/AKT signaling pathway via PTEN affected histone deacetylase 5 (HDAC5)-mediated EMT of renal tubular cells [231]. The PI3K/AKT/GSK-3β pathway can mediate oxidative stress and apoptosis in DKD. Using a mouse model of STZ-induced DKD, Wang et al. have demonstrated that phillyrin (a major active component of *Forsythia suspensa*) inhibited glycogen synthase kinase-3β (GSK-3β) activity by activating the PI3K/AKT signaling pathway, increased the Bcl-2/Bax ratio, reduced the release of cytochrome c from the mitochondria to the cytoplasm, subsequently inhibited the activation of caspase-3 and ultimately suppressed renal cell apoptosis [232]. The PI3K/AKT signaling pathway, in DKD, was also shown to regulate oxidative stress and inflammation by controlling GSK-3β/Nrf2 and ASK1/JNK signaling pathways, respectively [233]. Increasing evidence suggests that many signaling pathways in DKD have been implicated in AKT phosphorylation, and activation of AKT is required for DKD occurrence and development [234,235,236]. AKT, in conjunction with glycogen synthase kinase-3β (GSK-3β) as its downstream mediator, is instrumental in regulating mitochondrial functions [237]. Within a high glucose environment, mitochondrial dysfunction has a pivotal role in both the onset of oxidative burst and the initiation of apoptosis [238]. Recent studies have underscored the interrelation between AKT activation and mitochondrial function, oxidative stress, and apoptosis [239,240]. The phosphorylated form of AKT elicits an opposing regulatory influence on GSK-3β [236]. In the context of DKD, AKT phosphorylation is restrained, leading to the activation of GSK-3β [233]. Subsequently, this activated GSK-3β orchestrates the equilibrium between Bax and Bcl-2, thereby influencing mitochondrial permeability. This instigates the opening of the mitochondrial permeability transition pore, culminating in the liberation of cytochrome C from the mitochondria, ultimately contributing to the regulation of cell apoptosis [241,242]. 

Recent studies focused on JAK/STAT signaling shed light on its involvement in various factors linked to the progression of DKD including fibrosis, immunity, inflammation, aging, autophagy, and EMT. Thus it stands as the central signaling hub in the advancement of DKD [243]. A direct relationship between tubulointerstitial JAK/STAT expression and progression of kidney failure in patients with DKD has been observed [244]. The activation of JAK/STAT signaling pathways has been implicated in tubulointerstitial fibrosis and epithelial to mesenchymal transition in various conditions, including DM, as demonstrated in animal models [245,246,247]. Additionally, JAK/STAT activation has been observed in rat glomerular cells exposed to high glucose [248,249]. In the STZ-induced DKD mouse, findings illustrate that elevated glucose levels lead to the activation of JAK2 and the STATs, primarily through an AngII-dependent mechanism leading to the initial kidney damage [248]. Furthermore, elevated glucose levels stimulate the proliferation of glomerular mesangial cells (GMC) and trigger the production of TGF-β. Concurrent incubation with AG-490, a specific JAK2 inhibitor, effectively counteracted the high glucose-induced surge in TGF-β and fibronectin synthesis. AG-490 also nullified the tyrosine phosphorylation of JAK2, STAT1, and STAT3 induced by high glucose in GMC. When GMC cultured in 25 mmol/L glucose were preincubated with a specific JAK2 or STAT1 antisense oligonucleotide, both TGF-β and fibronectin synthesis were prevented. These findings offer direct evidence of the connections between JAK2, STAT1, and the excessive production of TGF-β and fibronectin induced by glucose in GMC [249]. Moreover, in the study by Zheng et al., it was discovered that suppressing STAT3 resulted in an enhancement of renal function and a reduction in the expression of TGF-β1, VEGF, and other factors associated with fibrosis and collagen buildup in C57BL/6 diabetic mice [250].

The Notch signaling pathway in the mammalian system comprises four transmembrane receptors (Notch1–Notch4), two Jagged family ligands (JAG1 and JAG2), and three delta-like ligands (DLL1, DLL3, and DLL4) [191,251]. Activation of the Notch signaling pathway was observed in patients with tubular interstitial fibrosis (TIF) as well as in TIF mouse models. Additionally, it was established that the Notch pathway is both necessary and sufficient for the initiation and progression of TIF [191]. In a mouse model of DKD, it was demonstrated that gliquidone, a diabetic medication, can ameliorate the diabetic symptoms of DN by inhibiting the Notch/Snail1 signaling pathway, improving anti-oxidative response, and delaying renal interstitial fibrosis in a dose-dependent manner [252]. In another study by Nishad et al., both in vitro using immortalized human podocytes and in a mouse model, it was shown that excess growth hormone (GH) activates Notch1 signaling in a γ-secretase-dependent manner. Pharmacological inhibition of Notch1 using the γ-secretase inhibitor DAPT (N-[N-(3,5-Difluorophenacetyl)-l-alanyl]-S-phenyl glycine t-butylester) resulted in reduced podocyte loss. Importantly, the results demonstrated that DAPT treatment halted cytokine release and prevented glomerular fibrosis, all of which were induced by excess GH. Furthermore, DAPT prevented GH-induced GBM thickening and proteinuria [253]. Moreover, the Notch signaling pathway may regulate oxidative damage and apoptosis in glucose-mediated renal tubular epithelial cells by controlling mitochondrial dynein and biogenesis genes, potentially accelerating renal interstitial fibrosis in DKD [254]. 

### 5.5. Mineralocorticoid Receptor (MR) and Aldosterone

Mineralocorticoids are a class of steroid hormones, with aldosterone being the primary physiological mineralocorticoid. It is produced in the outer layer of the adrenal gland as a response to hyponatremia and hyperkalemia, triggered by the activation of the RAAS [255]. There is growing evidence indicating the existence of an intricate network involving aldosterone, the MR, and Ras-related C3 botulinum toxin substrate 1 (Rac1) as crucial elements in the generation of ROS and subsequent damage caused by oxidative stress. This dynamic interaction plays a significant role in initiating interstitial nephritis, ultimately culminating in fibrosis in cases of DKD [256]. 

The MR functions as intracellular receptors, operating as a nuclear transcription factor or exerting rapid non-genomic effects through secondary cell signaling pathways [257,258]. The MR was initially believed to exclusively bind with mineralocorticoids, but subsequent research revealed that aldosterone, cortisol, and progesterone bind to the MR with equal affinity [259]. The MR instigates the inflammatory cascade by generating ROS through NADPH in the mitochondria, a process further amplified by Rac1 [260]. Additionally, aldosterone augments the expression and activation of serum- and glucocorticoid-inducible protein kinase 1 (SGK1), a factor associated with the development of renal fibrosis [260]. In a study using uninephrectomy in type 2 diabetic mouse models, it was It was demonstrated that salt-induced activation of the Rac1-MR pathway in distal tubules and glomeruli played a role in DKD progression through hypertension and glomerular injury, respectively. This discovery underscores the potential of MR antagonism in conjunction with Rac1 inhibition as a novel strategy for DKD treatment [261]. Both Rac1 and aldosterone contribute to fibrosis by activating inflammasomes. Studies indicate that exposing macrophages to elevated levels of aldosterone leads to inflammasome activation via mitochondria-derived ROS. This inflammasome activation in macrophages has been shown to mediate aldosterone-induced renal fibrosis in a murine model [262]. The expression of a well-studied inflammasome, NLRP3, was shown to be heightened in the glomeruli of DKD patients, correlating with the degree of albuminuria [263]. Within podocytes, aldosterone, acting through an MR, in conjunction with Rac1, triggers the activation of the NLRP3 inflammasome, resulting in podocyte injury and glomerular sclerosis [264].

**Figure 6:**
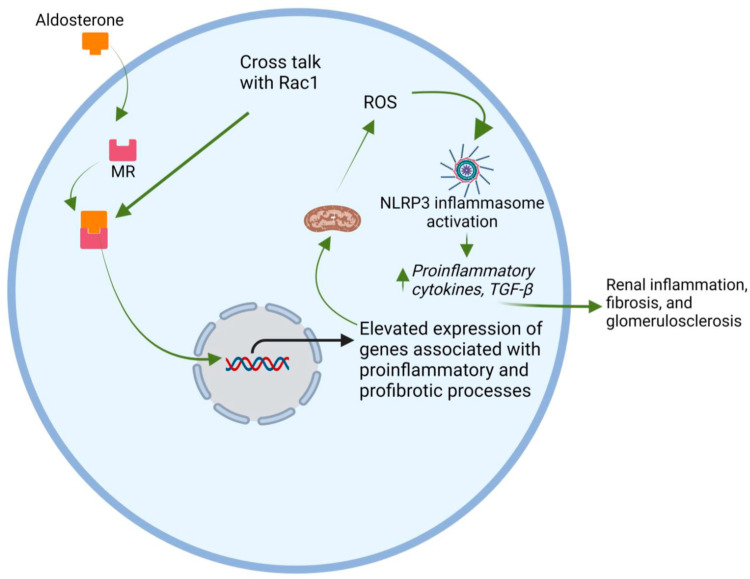
Mineralocorticoid receptor (MR) and aldosterone in DKD. The diagram depicts the involvement of the MR and aldosterone in the development of DKD. The MR functions as an intracellular receptor, instigating the inflammatory cascade with aldosterone by generating ROS in the mitochondria, a process further amplified by Rac1. Both Rac1 and aldosterone contribute to fibrosis and inflammation by activating inflammasomes, NLRP3. The activation of the NLRP3 inflammasome, results in renal inflammation, fibrosis, and glomerular sclerosis. Abbreviations: DKD: diabetic kidney disease, MR: mineralocorticoid receptor, ROS: reactive oxygen species NLRP3: nucleotide-binding domain, leucine-rich-containing family, pyrin domain-containing-3.

Aldosterone triggers the activation of inflammasomes in macrophages, provoking an inflammatory tubulointerstitial region [262]. Additionally, macrophages play a crucial role in generating renal TGF-β, a key factor in renal fibrosis [265]. The pathway leading to TGF-β-driven renal fibrosis seems to be facilitated by Rac1, which acts as a substantial redox-dependent non-SMAD (noncanonical) regulatory factor [265]. The role of the MR and aldosterone is depicted in Figure 6.

While it is acknowledged that aldosterone exhibits MR-independent effects contributing to DKD development, and Rac1 can be upregulated without MR activation, MR remains a pivotal component in this interaction. Clinical evidence supports the efficacy of the novel non-steroidal MR antagonist (nsMRA), finerenone, in slowing the progression of DKD [256].

### 5.6. OPN-Mediated Fibrosis in DKD

**Figure 7:**
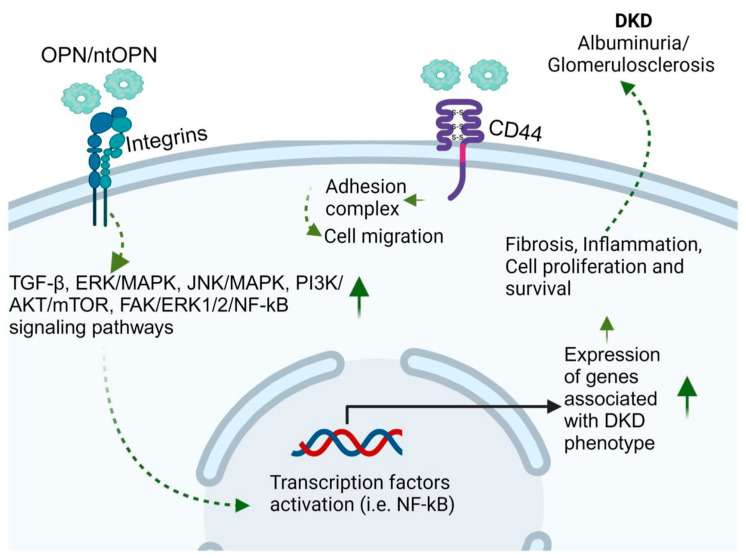
OPN-mediated fibrosis in DKD. The diagram illustrates OPN-mediated signaling pathways. The detrimental impacts of OPN occur via its interaction with receptors, integrins, and CD44. Binding to these receptors leads to significant proinflammatory functions, enabling OPN to trigger the activation of various pathways, including cell survival, cell proliferation, angiogenesis, migration, and fibrosis. Abbreviations: OPN: osteopontin, ntOPN: N-terminal osteopontin, TGF-β: transforming growth factor-β, ERK/MAPK: extracellular signal-regulated kinase/mitogen-activated protein kinase, JNK/MAPK: jun N-terminal kinase/mitogen-activated protein kinase, PI3K/AKT/mTOR: phosphatidylinositol-3-kinase/Ak strain transforming/mammalian target of rapamycin, FAK/ERK1/2/NF-κB: focal adhesion kinase/extracellular signal-regulated kinase 1 and 2/nuclear factor kappa B.

OPN is a secreted, pleiotropic, multi-phosphorylated glycoprotein, first recognized as secreted phosphoprotein 1 (SPP1) in 1979 [266]. The kidneys possess the highest concentration of OPN compared to other tissues [267]. During kidney injury, its presence is significantly increased in all tubular segments and especially in the glomeruli [268]. The role of OPN in DKD has been reviewed in detail by our group recently [269]. During the last decade, a number of studies analyzed the role of OPN in the pathogenesis of DKD and reported high expression of OPN in the tubular epithelium of the renal cortex and in glomeruli in rat and mouse models of DKD [270,271]. Our lab has shown that TGF-β likely mediates the effect of OPN in DKD mouse models enhancing glomerular damage [14]. In this study, we generated STZ-induced experimental, and genetic models of type 1 (Ins2*^Akita^*) and type 2 (Lepr*^db^*^/*db*^) diabetic mice on the background of OPN-null and wild-type mice. In both mouse models, OPN deletion decreased albuminuria, glomerular mesangial area, fractional volume of expansion, and expression of glomerular collagen IV, fibronectin, and TGF-β in the diabetic mice compared with their respective controls. In in vitro experiments with cultured mouse mesangial cells, the TZDs, rosiglitazone and pioglitazone, but not insulin, decreased AngII-induced OPN expression, while recombinant OPN upregulated TGF-β, ERK/MAPK, and JNK/MAPK signaling, which have been shown to be involved in the pathology of DKD. These studies strongly suggested that OPN expression enhanced glomerular damage, likely through the expression of TGF-β [14]. Our findings gained further support from a recent investigation in the German Chronic Kidney Disease cohort [272]. This study revealed a correlation between elevated OPN levels and a deterioration in kidney function markers, along with an increased likelihood of experiencing adverse outcomes. The researchers concluded that a significant portion of kidney function decline could be attributed to heightened OPN levels [272]. The full-length OPN protein is cleaved by various proteases, including thrombin, matrix metalloproteinase (MMP)-3, MMP-7, cathepsin-D, and plasmin, producing ntOPN, which may have more detrimental effects in CKD [269]. Therefore, we further investigated whether ntOPN may be a better predictor of DKD, and this hypothesis was subsequently tested in a Chinese population [15]. Collectively, our research showed that urinary ntOPN stands out as an independent marker for both the initiation and the progression of DKD. When compared to conventional biomarkers like serum creatinine combined with urinary albumin-to-creatinine ratio, our multi-biomarker models centered around urinary ntOPN, demonstrated enhanced predictive power in forecasting DKD progression. This development holds the potential to provide a more accurate biomarker for evaluating the risk of DKD in individuals with DM [15]. Another study, although in a different scenario, demonstrated that milk ntOPN bounded intestinal cells most effectively and was transported across the intestinal membrane indicating that proteolytic processing of OPN to form ntOPN increases its biological activity [273]. These findings support the efficacy of OPN/ntOPN as a therapeutic target in DKD. The OPN-mediated signaling pathways are shown in Figure 7.

## 6. Genetics and Epigenetics of DKD

Studies have demonstrated a familial clustering of DKD across diverse ethnic groups, suggesting a genetic contribution to its development. Additionally, genetic risk factors in DKD interact with environmental elements such as lifestyle, diet, and medication. The interplay between genetic, epigenetic, and environmental factors in the initiation and progression of DKD has been illustrated in several studies [274,275,276,277,278]. Genetic investigations of DKD primarily focus on analyzing associations between variations in genomic DNA, such as single nucleotide polymorphisms (SNPs), copy number variants (CNVs), and microsatellites, with the clinical manifestations of the disease [274,276,279,280]. Notably, significant linkage peaks have been observed on chromosomes 3q and 18q, implicating genes like AngII receptor type 1 [281], adiponectin [282], non-catalytic region of tyrosine kinase adaptor protein 1 (NCK1) [283], and carnosine dipeptidase 1 (CNDP1) [284] in DKD susceptibility. Recently, a meta-analysis of genome-wide association studies (GWAS), including 19,406 individuals of European ancestry with type 1 DM was performed by Salem et al. Within this analysis, they found 16 risk loci of genome-wide significance. Notably, the most strongly associated variant (rs55703767) is a prevalent missense mutation found in the collagen type IV alpha 3 chain (COL4A3) gene that encodes a vital structural element of the glomerular basement membrane [285]. In a separate investigation involving 13,123 individuals with type 2 DM, a meta-analysis uncovered a fresh link between DKD and the variant rs72763500 (located at chr1:236116561). This variant serves as a splicing quantitative trait locus for the nidogen-1 (NID1) gene. NID1, a significant constituent of the basement membrane in renal tubules, potentially exerts an influence on the development of DKD in T2D [286]. Further, in a thorough GWAS study mega-analysis encompassing 33,879 patients, scientists pinpointed particular SNPs (specifically, rs3128852, rs117744700, and rs28366355) linked to DKD. Importantly, they confirmed the causative connection between rs3128852 and the onset of DKD [287]. Through GWAS of the Family Investigation of Nephropathy and Diabetes cohort, we have identified several novel loci associated with albuminuria, eGFR, DM, DKD, advanced DKD, and renal failure [288,289,290,291,292,293]. This discovery not only elucidated the genetic landscape of DKD but also paved the way for further exploration through functional analyses. Additionally, it offered new prospects for developing innovative diagnostic markers for DKD. Hence, these studies represented progress toward a broader comprehension of the underlying mechanisms of DKD. 

On the other hand, in the realm of DKD, epigenetic investigations focus on potentially heritable changes in gene expression that arise without any alterations in the underlying DNA sequence [277,294,295]. These studies reveal how environmental factors can influence the patterns of gene expression implicated in the progression of DKD. For instance, a comprehensive analysis of methylomes, transcriptomes, and genetic variations in 500 patients with DKD identified 40 loci displaying altered methylation and expression strongly tied to DKD. These loci demonstrated functional connections to processes like complement activation, inflammation, and apoptosis [296]. Another study identified 19 CpG sites linked to eGFR, including five associated with confirmed renal fibrosis, indicating DNA methylation alterations in the kidney cortex [297,298]. Additionally, abnormal DNA methylation patterns were noted in proximal tubular epithelial cells from the murine model of DM, with genes functionally associated with mitochondrial biogenesis [299]. The interplay between gene methylation levels and clinical markers of DKD is significant [300,301]. For example, elevated albuminuria correlated with hypomethylation of tissue inhibitor metalloproteinase-2 (TIMP-2) and aldo-keto reductase family 1 member B [302], while higher serum homocysteine levels were observed in DKD patients with increased promoter methylation of the methylenetetrahydrofolate reductase gene [303]. CTGF, associated with extracellular matrix accumulation and DKD pathogenesis, exhibited lower DNA methylation levels and increased protein levels, correlating with albuminuria and eGFR decline in DKD patients [304]. Furthermore, the RAS protein activator like 1 (Rasal1) gene, displayed hypermethylation of its promoter in DKD, contributing to fibroblast activation and kidney fibrosis progression [305]. Changes in Rasal1 promoter DNA methylation were reversed by tet methylcytosine dioxygenase 3-mediated hydroxymethylation, with a concomitant reduction in fibrosis [305]. In summary, these findings emphasize the dynamic regulation of multiple signaling pathways by DNA methylation in the progression of DKD. 

**Figure 8:**
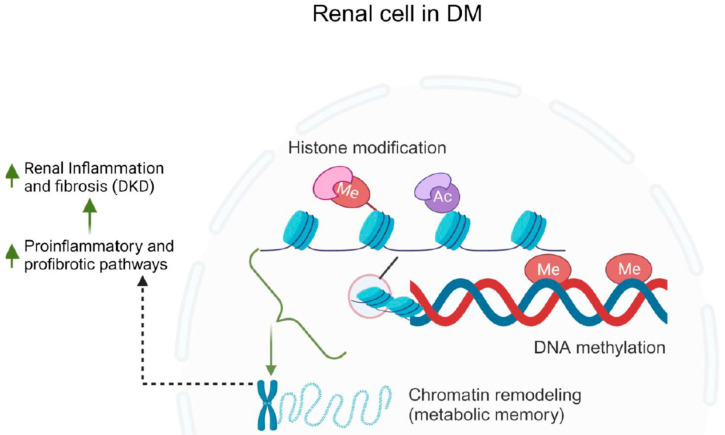
Epigenetics in DKD. Histone modifications, specifically acetylation and methylation, along with DNA methylation, are linked to the aberrant regulation of genes associated with inflammation and fibrosis in DKD. Abbreviations: DM: diabetes mellitus, DKD: diabetic kidney disease, Me: methylation, Ac: acetylation, DNA: deoxyribonucleic acid.

In the context of DKD, histone modifications are crucial in regulating gene expression patterns that contribute to the progression of the disease [306,307]. For instance, studies have shown that certain histone modifications, such as acetylation and methylation, are associated with the dysregulation of genes involved in inflammation [308,309,310,311], fibrosis [312,313], and oxidative stress responses [314,315] in DKD. These modifications create a molecular environment that promotes the activation of pro-inflammatory and pro-fibrotic pathways, ultimately leading to the structural and functional changes observed in diabetic kidneys. Histone methylation occurs at arginine, lysine, and histidine sites located on histone tails [316] that can either enhance or inhibit gene transcription [311]. Lysine methylation is regulated by enzymes known as methyltransferases (KMTs) and can be reversed by demethylases (KDMs) [317,318,319]. One category of KMTs includes enzymes containing SET domains [317]. In diabetic mice, there was an increase in KDM6A expression and a decrease in H3K27me2/3 levels observed in the kidneys [320]. This pattern was also evident in podocytes and renal tubular cells subjected to hyperglycemic conditions [321,322]. Moreover, kidney tissues from individuals with DKD displayed reduced levels of H3K27me3 in podocytes, glomerular cells, and tubular cells [323]. Comparatively, diabetic animals exhibited elevated levels of H3 lysine 4 mono-methylation (H3K4me1) and H3K4me3, along with decreased levels of H3K27me3 in the kidneys when compared to non-diabetic controls [324]. Moreover, being exposed to high blood sugar levels early in life, particularly during fetal development, heightens the risk of developing type 2 DM in adulthood [325]. There is mounting evidence suggesting that prolonged hyperglycemia induces a “metabolic memory” through epigenetic alterations in DKD, which alter gene expression [311,325]. The role of epigenetics in DKD is summarized in Figure 8.

## 7. Biomarkers Associated with DKD 

In DKD, biomarkers offer valuable insights into the early detection, progression, and management of the condition, enabling timely interventions to slow or halt its progression. Additionally, they facilitate research efforts aimed at understanding the underlying mechanisms of DKD and developing innovative therapies. Overall, biomarkers serve as indispensable tools in the fight against DKD, contributing to improved patient outcomes and quality of life. There are several important DKD biomarkers that have been studied, as shown below in Table 1.

### 7.1. Biomarkers Associated with Tubular Damage in DKD

Several biomarkers for tubular damage in DKD have been identified. For example, plasma kidney injury molecule-1 (KIM-1) is a notable indicator of early tubular injury and stress in DKD [176,326,327]. Markers like TNFR-1 and TNFR-2, receptors for tumor necrosis factor [176,326,328], along with neutrophil gelatinase-associated lipocalin (NGAL) [327,329,330] provide insights into acute kidney injury and inflammation, aiding in the evaluation of tubular damage. The urinary liver-type fatty acid-binding protein (L-FABP) is specific to proximal tubules and its presence in urine indicates early tubular damage [331,332]. OPN [14] and ntOPN [15], a protein associated with kidney injury, contribute to understanding tubular damage in DKD. Urinary AngII-converting enzyme 2 (ACE2), a component of the renin–angiotensin system, and angiotensin, known for their roles in blood pressure regulation, also offer valuable indicators of tubular health [333,334]. Additionally, markers such as n-acetyl-β-D-glucosaminidase (NAG) [334,335], α1-microglobulin [336,337], fibroblast growth factor 23 (FGF-23) [338], α-klotho [339], and a disintegrin and metalloprotease-10 (ADAM-10) [340] play pivotal roles in assessing tubular function and damage. This comprehensive array of biomarkers provides a multifaceted approach to evaluating tubular health and damage in individuals with DKD. 

### 7.2. Biomarkers Associated with Glomerular Damage in DKD 

Similarly, biomarkers of glomerular damage play a critical role in evaluating DKD. Transferrin, a glycoprotein, has shown promise as a potential biomarker, as elevated levels have been associated with glomerular dysfunction in DKD [341,342,343]. Type IV collagen, a structural protein in the basement membrane of glomeruli, is another key biomarker [344,345]. Elevated levels of type IV collagen indicate basement membrane thickening and glomerular damage in DKD. Cystatin C, a protein freely filtered by the glomerulus, offers insights into the glomerular filtration rate and serves as an indicator of glomerular health [346,347]. Ceruloplasmin, an enzyme involved in iron metabolism, has also been implicated in DKD and may serve as a biomarker for glomerular damage [342]. Fibronectin, an adhesive glycoprotein, is associated with glomerular injury and fibrosis [348]. Urinary podocytes–podocalyxin [349], and vascular endothelial growth factor (VEGF) [337] predict the progression of the disease and are shown to significantly correlate with urinary albumin excretion. Additionally, α-klotho [350], and ADAM-10 [340,351] play crucial roles in glomerular health and function. Also, urinary proteomics identifies cathepsin D as a biomarker of rapid eGFR decline in type 1 diabetes [352]. Together, these biomarkers provide a comprehensive toolkit for assessing glomerular health and damage in individuals with DKD.

Recently, urine levels of adenine, a metabolite produced in the kidney, was shown to be predictive and a causative biomarker of looming progressive kidney failure in patients with DM, a finding that could potentially lead to earlier diagnosis and intervention. The elevated adenine was also associated with all-cause mortality [353]. This was the first study to also show that an inhibitor of endogenous adenine was nephroprotective.

### 7.3. Inflammatory and Oxidative Stress Biomarkers of DKD 

Inflammatory and oxidative biomarkers are additionally pivotal in DKD development. Elevated markers like TNF-α signify progression to severe stages, including ESRD and GFR loss [354]. MCP-1 predicts renal disease progression, correlating with albuminuria and exacerbating inflammation and fibrosis [355]. TGF-β is linked to higher mortality risk and macroalbuminuria [193] while interleukins (IL-1β, IL-6, IL-8, IL-18) predict early renal decline risk [356]. These biomarkers highlight chronic inflammation in DKD. Likewise, oxidative stress, indicated by markers like 8-hydroxydeoxyguanosine (8oHdG) [357], pentosidine [358], uric acid [359], malondialdehyde (MDA) [360], superoxide dismutase (SOD) [360], and glutathione peroxidase (GPx) [361], contributes significantly to DKD. The interplay of inflammation and oxidative stress worsens kidney injury. Understanding and monitoring these biomarkers offer crucial insights into DKD mechanisms, suggesting potential therapeutic targets.

**Table 1:** Renal biomarkers associated with DKD.

Tubular	Glomerular	Inflammatory/Oxidative Stress
KIM-1, TNFR-1, and TNFR-2 [176,326,327,328]	Transferrin [341,342,343]	TNF-α [354]
NGAL [327,330]	Type IV collagen [344,345]	MCP-1 [355]
Urinary L-FABP [331,332]	Cystatin C [346,347]	TGF-beta [193]
OPN [14], ntOPN [15]	Ceruloplasmin [342]	ILs [356]
ACE2 [333,334]	Fibronectin [348]	8oHdG [357]
NAG [334,362]	Podocytes-podocalyxin [349]	Pentosidine [358]
α1-microglobulin [336,337]	VEGF [337]	Uric acid [359]
FGF-23 [338]	α-alpha-Klotho [350]	MDA [360]
α-Klotho [339]	ADAM-10 [340,351]	SOD [360]
ADAM-10 [340]	Cathepsins [352]	GPx [361]
Adenine [353]	Adenine [353]	

**Abbreviations:** KIM-1: kidney injury molecule-1, TNFR: TNF-α receptor, NGAL: neutrophil gelatinase-associated lipocalin, L-FABP: liver-type fatty acid-binding protein, OPN: osteopontin, ntOPN: N-terminal OPN, ACE2: angiotensin-converting enzyme 2, NAG: N-acetyl-β-D-glucosaminidase, FGF-23: fibroblast growth factor 23, ADAM-10: a disintegrin and metalloprotease 10, VEGF: vascular endothelial growth factor, MCP-1: monocyte chemoattractant protein-1, TGF-β: transforming growth factor-β, ILs: interleukins, 8-Ohdg: 8-hydroxydeoxyguanosine, MDA: malondialdehyde, SOD: superoxide dismutase, GPx: glutathione peroxidase.

## 8. Therapies Targeting Specific DKD Pathomechanisms 

Continuous understanding of the mechanisms involved in the development of DKD has led to the development of several pathomechanism-targeted therapies over the past two decades. For instance, until recently, the mainstay of therapy for DKD highlighted metabolic and hemodynamic-related therapies via optimization of glucose and blood pressure control and the use of RAS inhibitors, namely ACE inhibitors and ARBs. Despite these measures, significant residual risk for DKD progression remained, which then led to the discovery of newer therapies that have emerged as both reno- and cardio-protective agents. In particular, SGLT2 inhibitors, function as another hemodynamic target, and nsMRAs, interrupt aldosterone signaling via the mineralocorticoid receptor, which undergoes over activation in DKD, have provided significant hope and continue to extend kidney life and the overall lifespan for patients with DKD. A summary of the available treatment options in DKD is presented in Table 2.

### 8.1. RAAS Inhibitors

The Kidney Disease Improving Global Outcomes (KDIGO) as well as the American Diabetes Association (ADA) guidelines have recommended the use of ACE inhibitors and ARBs for patients with DKD to reduce blood pressure and to achieve ≥30% reduction in albuminuria [363,364,365]. In addition to their hemodynamic effects, described above, ACE inhibitors and ARBs can block oxidative stress and pro-inflammatory pathways. Despite their renal benefit to reduce DKD progression observed from large kidney outcome trials, Reduction of Endpoints in NIDDM with the Angiotensin II Antagonist Losartan [125] and the Irbesartan Diabetic Nephropathy Trial [136], ACE inhibitors and ARBs have demonstrated no evidence to reduce the composite of cardiovascular endpoints (cardiovascular death, myocardial infarction, hospitalization for heart failure, hospitalization for angina or lower limb amputation above the ankle). 

### 8.2. SGLT2 Inhibitors

The individual KDIGO and ADA guidelines as well as the KDIGO-ADA consensus report have recommended the use of SGLT2 inhibitors in patients with DKD and cardiovascular disease on top of standard of care to address the residual risk of DKD progression. These new therapies function through the reduction of tubuloglomerular feedback and glomerular hyperfiltration, as we described above [363,364,365]. Notably, the broad renal and cardiovascular benefits of this new class of drugs may not be fully explained through this mechanism. Emerging evidence suggests that SGLT2 inhibitors may function via other mechanisms including blockade of the SGLT2 nutrient surplus sensor capacity [366,367] as well as other pleiotropic mechanisms [368]. For example, the inhibition of SGLT2 acts as a protective measure against organ hypoxia, a pathway frequently linked to the progression of DKD due to its role in fostering and sustaining fibrotic and inflammatory responses [369,370]. Additionally, studies on human proximal tubular cells, murine models of type 2 DM, and patients with type 2 DM have revealed that the inhibition of systemic SGLT2 results in a reduction of various molecules associated with inflammation, extracellular matrix turnover, and fibrosis. These molecules include TNFR1, MMP-7, IL-6, and fibronectin 1 [371,372,373,374]. A large meta-analysis of placebo-controlled trials indicates that the use of SGLT2 inhibitors modifies the risk of CKD progression and acute kidney injury, not only in patients with type 2 DM at high cardiovascular risk but also in patients with CKD or heart failure irrespective of DM [375]. 

### 8.3. nsMRAs

The nsMRAs have emerged as novel anti-inflammatory and anti-fibrotic therapies with proven efficacy to reduce the risk of renal function decline as well as ESRD, cardiovascular death, non-fatal heart attacks, and hospitalization for heart failure. While finerenone is the first-in-class nsMRA approved for clinical use, there are several other selective nsMRAs with anti-inflammatory and anti-fibrotic properties undergoing assessment through clinical trials [376,377].

### 8.4. ET Receptor Antagonist

ET, a polypeptide, is known as a potent vasoconstrictor [378,379]. It is produced by epithelial and mesangial cells in the kidney and plays a crucial role in regulating blood flow, and glomerular filtration, as well as water, sodium, and acid-base balances [379,380,381]. ET exerts its effects through ET receptors A (ETA) and B (ETB), expressed on various renal components including glomerular podocytes, mesangial cells, and arterioles [379,380,381]. ET’s activation of these receptors can have detrimental effects on the kidney, contributing to the progression of DKD [382,383,384]. Therefore, blocking ET receptors with ET receptor antagonists (ERAs) like Atrasentan, has been shown to have protective effects on the kidneys in patients with DM [385]. ERAs impact glomerular hemodynamics, leading to improved blood pressure, reduced proteinuria, and a balanced filtration rate [382,386]. They also provide kidney protection by addressing issues like podocyte injury [382,387], reducing mesangial matrix accumulation [388], and mitigating fibrosis and inflammation [389,390]. This leads to a decrease in glomerular permeability and proteinuria [391]. Nonetheless, findings from clinical trials suggest that these substances can also lead to side effects related to fluid volume [391,392]. Combining ERAs with SGLT2 inhibitors or utilizing dual Agn-II type 1/ET receptor blockers have been suggested as strategies to overcome these challenges [379,393].

**Table 2:** Summary of the available medications to treat patients with DKD.

Targeted Pathways	Therapeutic Function	Clinical Outcomes	References
RAAS Blockers	Reducing inflammatory and fibrotic processes	Slow the deterioration of kidney function. Reduce the risk for creatinine doubling, and ESRD.	[125,136,394,395]
SGLT2 inhibitors	Blocking reabsorption of glucose in the proximal tubule	Minimize significant eGFR decline, kidney failure, heart failure, and mortality due to kidney and CVD.	[372,396,397,398,399]
Nonsteroidal mineralocorticoid receptors (MRs) antagonist	Downregulating inflammatory and fibrotic pathways in nonepithelial cells; Downregulating ion channels for sodium and potassium in kidney tubular epithelial cells	Minimizes significant eGFR decline, kidney failure, heart failure, and mortality due to CKD.	[400,401,402]
Endothelin receptor antagonist	Downregulating inflammatory and fibrotic pathways; Efferent artery vasodilation	Reduces albuminuria, eGFR decline, kidney failure as well as death from CKD.	[385,403]

**Abbreviations:** RAAS: renin–angiotensin–aldosterone system, ESRD: end stage renal disease, SGLT2: sodium–glucose cotransporter 2, eGFR: estimated glomerular filtration rate, CVD: cardiovascular disease, MR: mineralocorticoid receptor CKD: chronic kidney disease.

## 9. Conclusions and Future Directions

There remains a notable gap in our understanding of DKD that warrants further research. While significant progress has been made in identifying key factors and biomarkers, understanding the underlying molecular mechanisms, and developing targeted therapies, there is still much to uncover. One area of interest is the intricate interplay between genetic predisposition and environmental factors in the development and progression of DKD. The future of managing DKD encompasses a comprehensive approach. This involves tailoring therapies based on an individual’s genetic, molecular, and clinical profile. In DKD, maintaining precise glycemic control is essential, coupled with innovative approaches targeting inflammation and fibrosis. The modulation of the RAAS pathway holds particular significance. Additionally, the exploration of medications like SGLT2 inhibitors, and nsMRAs, as well as GLP-1 receptor agonists have shown considerable promise. Furthermore, exploring the potential benefits of combination therapies, such as the use of ERAs in conjunction with SGLT2 inhibitors, holds potential for improving outcomes in DKD patients. Moreover, long-term studies assessing the efficacy and safety of emerging treatments are essential to establish their viability in clinical practice. As research continues, new knowledge is gained, increasing the likelihood of identifying innovative targets that may potentially and completely eliminate the risk of CKD progression.

Early biomarker discovery and advances in regenerative medicine are emerging focal points in this field. Notably, our lab is actively researching ntOPN as a potential early biomarker and therapeutic target with the capacity to significantly reduce fibrosis and inflammation in DKD. In tandem with these pharmacological strategies, integrated care, and patient education are pivotal. Encouraging diversity in the participation of clinical trials is crucial and should be promoted. Ultimately, a comprehensive strategy integrating pharmacotherapy, personalized medicine, and cutting-edge technologies aims to improve outcomes and enhance the quality of life for individuals with DKD.

## Data Availability

Not applicable.
